# Supplementary material for: Effect of a dietary intervention including minimal and unprocessed foods, high in natural saturated fats, on the lipid profile of children, pooled evidence from randomized controlled trials and a cohort study
Source: PLoS One. 2022 Jan 5;17(1):e0261446. doi: 10.1371/journal.pone.0261446 (PMC8730453; doi:10.1371/journal.pone.0261446)
Supplement: Attachment — Submitted filename: Rebuttal Letter.docx [file pone.0261446.s003.docx]

**Rebuttal Letter 04-07-2021**

*PONE-D-21-01126*

*Effect of a dietary intervention including minimal and unprocessed foods, high in natural saturated fats, on the lipid profile of children, pooled evidence from randomized controlled trials and a cohort study.*

Dear Editors,

We would like to thank the reviewers for their time and valuable comments on the manuscript titled ‘Effect of a dietary intervention including minimal and unprocessed foods, high in natural saturated fats, on the lipid profile of children, pooled evidence from randomized controlled trials and a cohort study’. The input given by the reviewers definitely helped to improve the manuscript. Point-by-point responses to the reviewers’ comments in blue are attached below.

Major improvements were made to the reporting of methodological aspects of the study. In particular, refinement of the analytical methods. Secondly, the results section is improved since the update in the analytical methods was applied.

We believe that the manuscript is now suitable for publication in PLOS ONE. We hope you feel the same.

Kind regards,

Rosanne Hendriksen, MSc

[rosanneh@live.nl](mailto:rosanneh@live.nl)

Ellen van der Gaag, Pediatrician MD, PhD

Hospital Group Twente, Hengelo, The Netherlands

[e.vdgaag@zgt.nl](mailto:e.vdgaag@zgt.nl)

Reviewer #1: Specific comments

1) Additional information on the composition of the diet (specific foods, macronutrient composition, fatty acid composition, fiber, plant sterols/stanols) that control subjects consumed as well as the level of physical activity would be useful, not only for the present report but for comparison with previous and future studies.
2) Additional information on the foods (macronutrient composition, fatty acid composition, fiber, plant sterols/stanols) consumed by subjects in the intervention group besides the 4 foods listed in Table 1 as well as the level of physical activity would also strengthen the manuscript.

We agree that additional information on the composition of the diet and physical activity level would definitely strengthen the manuscript. Unfortunately, this additional information is partially available for both the control group and intervention group. Therefore, this was not mentioned in the manuscript. Physical activity is an interesting aspect, but was not the focus of our study. It will be interesting to study it in follow up studies. We have mentioned it in the discussion.

3) The manuscript indicates that normality was checked by the Shapiro-Wilk test, but was normality found? If not, what action was taken?

Normality was indeed checked by Shapiro-Wilk test. Normality was found only by LDL cholesterol at follow-up and by total cholesterol at follow-up. The rest of the measurement were not normally distributed. This is added to the manuscripts.

4) The manuscript indicates that “All of these external factors did not affect the lipid profile of the children significantly (data not shown)”. The results of these statistical tests should be included in the manuscript whether statistically significant or not.

We removed this sentence, since it was confusing and didn’t add any additional information to the manuscript.

5) The type and amounts of beverages, notably the caloric ones, consumed by the intervention and control subjects should be documented in the manuscript if possible, and discussed.

Unfortunately, the information about the consummation of beverages is not available. Therefore, this is not documented in the manuscript. But this would also be an interesting topic for follow-up studies. This is mentioned in the discussion.

Reviewer #2:

**Title page:** the short title 'a pooled analysis' is insufficiently descriptive of this study; please suggest a more informative title. I would also suggest that the main title include the description that this is a pooled analysis e.g. by adding " - pooled evidence from randomised controlled trials and a cohort study".

The main title is changed to: Effect of a dietary intervention including minimal and unprocessed foods, high in natural saturated fats, on the lipid profile of children, pooled evidence from randomized controlled trials and a cohort study.

The short title is changed to: Minimal processed foods; pooled evidence on lipid profiles.

**Abstract:** The methods need a bit more detail e.g. one sentence to explain how you conducted the pooled analysis, since this is a key feature of this study. The results in the abstract also don't seem to make sense: for example, you say BMI increased from 0.023 to 0.15 - these are impossibly low values of BMI, unless you meant that these were the changes in BMI (but even then the sentence would still not make sense). Please recheck these. Lastly, the appropriate way to report results is to report the effect/difference, its 95% confidence intervals and the p-value for the hypothesis test of 'no-difference'.

- In the method part of the abstract this sentence was added: *This pooled analysis study consist of combines three intervention studies; one non-randomized retrospective cohort study and two randomized controlled trials, to a pooled analysis.* To clarify that this study combines 3 previous studies into one pooled analysis study.
- BMI levels discussed in this manuscript (both in the abstract and the results part) are SD BMI levels, but we sometimes called them ‘BMI levels’. To prevent discussion, we changed all BMI values into SD BMI.
- The results part of the abstract and the results part on the manuscript were both updated with 95% confidence intervals and p-values.

**Methods:** the current methods are not sufficiently detailed. The study setting, participants, interventions and outcomes are reasonably well reported, but the statistical methods are glossed over. You could add a bit more description, perhaps one or two sentences each, of the underlying studies, e.g. what their objectives were. For the methods, there are several things you need to add to improve the reporting. First, you need to report how you summarised the participant characteristics. This is typically reporting counts and proportions for categorical variables and means or medians with standard deviations or IQRs, respectively, for continuous ones. This should preferably be done separately for each data source (e.g. see Table 1 in <http://www.thelancet.com/journals/langlo/article/PIIS2214-109X(17)30484-9/fulltext)>. Secondly, for this analysis of a pooled dataset you can't simply resort to t-tests, there are statistical issues relating to within-study clustering of the contributing studies to deal with. You need an appropriate method to pool estimates from studies, and this could be done either through (1) analysing the datasets independently (e.g. analysis of covariance/baseline-adjusted models for the continuous outcomes) and then combining the study specific estimates (separately for each outcome) using an appropriate method such as inverse variance weighting to pool them, or (2) combining all the studies into a single dataset and then using a mixed effects ANCOVA/baseline adjusted model on the combined dataset for each outcome, with random effects across studies. Method (1) would be further enhanced by including a forest plot of the study-specific effects and the pooled effect similar to what would be done in a meta-analysis. Comparison of baseline characteristics between treatment groups is unnecessary, especially for randomised trials where baseline imbalance is not expected anyway.

- A small description of the objectives of the previous studies was added in the manuscript.
- The participant characteristic table has changed (table 2). Characteristics were separated by study and only the main variables were showed. One column was added with the combined characteristics, which is relevant for this pooled analysis. For the categorial variables counts and proportions were added. For the continues variables means with medians and IQR’s were added.
- A mixed model ANCOVA test was used to check changes between groups over time. This was updated in the method of the manuscript.

**Results**: see comment above about comparing baseline characteristics between groups. Furthermore, in table 2 you have also compared them in terms of dietary compliance, which is a feature of the intervention which is necessarily expected to be different - please remove the p-value column from this table. In Table 3 you should not even be including the baseline values in the comparison; as suggested above, you should use the baseline values only for adjustment in the ANCOVA model i.e. a model of the follow-up values with treatment group as the main explanatory variable and including the baseline value of the outcome as a covariate. Table 3 should then report the follow-up mean and standard error (not SD unlike the baseline table) for each arm, followed by the effect (i.e. the coefficient for treatment in the ANCOVA model described above) with its 95% confidence intervals and p-value. You can have additional columns for effect/95%CI/p-value if you report both unadjusted and adjusted effects.

- Baseline characteristics were not compared to each other but were listed separately for each data source (Table 2).
- Dietary compliance is with p-values are removed from table 2. The compliance is shortly mentioned in the results section.
- After liberation we’ve chosen to include baseline values in Table 3 since we want to show the effect of the diet over time. The baseline values are the reference values and therefore we found they are important to show. Baseline values were used in the ANCOVA model. 95% CI an p-values were updated.
