# Supplementary material for: Effect of a dietary intervention including minimal and unprocessed foods, high in natural saturated fats, on the lipid profile of children, pooled evidence from randomized controlled trials and a cohort study
Source: PLoS One. 2022 Jan 5;17(1):e0261446. doi: 10.1371/journal.pone.0261446 (PMC8730453; doi:10.1371/journal.pone.0261446)
Supplement: Attachment — Submitted filename: Rebuttal Letter.docx [file pone.0261446.s004.docx]

**Rebuttal Letter 29-09-2021**

*PONE-D-21-01126*

*Effect of a dietary intervention including minimal and unprocessed foods, high in natural saturated fats, on the lipid profile of children, pooled evidence from randomized controlled trials and a cohort study.*

Dear Editors,

We would like to thank the reviewer for the time and valuable comments on the manuscript titled ‘Effect of a dietary intervention including minimal and unprocessed foods, high in natural saturated fats, on the lipid profile of children, pooled evidence from randomized controlled trials and a cohort study’. Point-by-point responses to the reviewers comments in blue are attached below.

Major improvements were made to the reporting of the results. In particular, the tables from the result section were adjusted to a more meaningful layout. Some minor improvements were made in the abstract and materials en methods section.

We believe that the manuscript is now suitable for publication in PLOS ONE. We hope you feel the same.

Kind regards,

Rosanne Hendriksen, MSc

[rosanneh@live.nl](mailto:rosanneh@live.nl)

Ellen van der Gaag, Pediatrician MD, PhD

Hospital Group Twente, Hengelo, The Netherlands

[e.vdgaag@zgt.nl](mailto:e.vdgaag@zgt.nl)

**Comments of the Reviewer**

**ABSTRACT:**

- SD BMI is introduced suddenly in the abstract without explanation; this should be described. Is it the standardised body mass index? If so, say so and also explain how the standardisation was done.

Any statements about SD BMI were removed from the abstract, since we found that it did not have additional value. It was determined as a secondary study outcome so it is introduced later in the manuscript with explanation.

- the methods should summarise how the pooled analysis was conducted; there is nothing in the current abstract that gives the reader any sense of what this pooled analysis constituted: for example, did you combine all data into a single dataset and then conduct regressions looking at the associations between exposures and outcomes in this combined/pooled dataset? If so, a sentence saying exactly this should be included in the methods after the sentence that currently ends on line 52.

A sentence about how this pooled analysis was performed was added at the end of the method part in the abstract.

- the statements from the second half of line 46 to the end of line 47 pertaining to the numbers included in the analysis are results and should be moved to the results section of the abstract.

This part is moved to the results section of the abstract.

- I appreciate that the authors have included effect estimates, confidence intervals and p-values in the results. However there is a problem with the results as presented. The point estimate of the difference between intervention and control group of 0.20 would be expected to lie within the confidence intervals; so a confidence interval of 1.30 to 1.65 is not consistent with this point estimate. Additionally, the correct order to cite the numerical results is: estimate 95%CI p-value, not estimate p-value 95%CI as currently done on line 55-56. Lastly, the result at the end of line 56-57 should also include an estimate of the magnitude of increase with 95%CI and p-value. I do wonder though what the relevance is of reporting changes over time in the control group - it is the difference in difference over time between the intervention and control groups which would be important to report here.

> We have checked alle confidence intervals in the manuscript, and indeed the one mentioned by the reviewer was not correctly described. This was updated.

> The citation order was updated to the right order.

> Any statements about changes over time in the control were removed and a sentence about significant changes between the intervention group and control group over time was added.

**METHODS:**

- more clarity is needed on what SD BMI means, especially how the standardisation was done to obtain this outcome measure.

*‘Standard Deviation (SD) BMI scores of the children were the secondary outcome measurements. Since absolute BMI scores can’t be compared between different age groups in young children, SD scores were determined. BMI was calculated by dividing weight in kilograms by the square of height in meters. The SD BMI score was calculated on the basis of gender, age, height, and weight.‘*

This part was added to the methods. We hope that we have clarify the meaning of SD BMI and how this was determined.

- more clarity is needed on how the 'pooling' was done here: did you pool raw data from the studies into a single dataset then analyse them, or did you pool the estimates from the three studies to obtain pooled estimates?

The sentence *‘Raw data of the three intervention studies where combined into one single dataset for analysis’* is added in the last paragraph of the method section.

- in line 129 you say participants were divided into control group and intervention group without describing what those groups received. This statement as it serves no particular purpose (and you say nothing about the same for the other two studies), and you should probably remove it from this section, as you later describe the interventions from line 141.

The sentence was deleted.

- I think you should remove all statements about tests for normality; they are not useful, as they are extremely sensitive to very slight deviations from normality. In any case, normality of outcomes in not a requirement for regression analyses or ANOVA/ANCOVA (multivariate normality or normality of residuals is what is important). Furthermore, the statements about outcome measures in line 156 to 158 are results and would not be included in the methods even if they were relevant. Please remove these and the tests for normality.

Statements about tests for normality are deleted.

**RESULTS:**

- effects or differences should be reported in this format: effect or difference, 95% confidence interval, p-value.

The citation order is updated to the format mentioned by the reviewer.

- as noted in the abstract, it is not possible for the increase of 0.20 mmol/L to have a confidence interval that does not straddle/include 0.20; the lower and upper 95%CI of 1.30 to 1.65 do not include 0.20 so either the point estimate or the confidence interval is incorrect.

Same as for the abstract: We have checked all confidence intervals in the manuscript, and indeed the one mentioned by the reviewer was not correctly described. This was updated.

- it would be more meaningful to present the difference in pre-treatment and post-treatment values of the outcomes compared between the intervention and controls groups i.e. difference-in-difference analysis. To do this, table 1 should include the baseline means and SDs of each of the outcomes in each group. Table 2 should then include the mean and SE (not SD) of each outcome in each arm in the follow-up period, along with the baseline-adjusted differences between the groups with 95%CI and p-values. You may also further adjust for other relevant factors if desired.

The two tables in the results section were changed. The first table (table 2) now includes the means and SD of the measurements of the lipid profile from the start of the study (baseline). The second table (table 3) includes the mean and SE of the measurements of the lipid profile from the follow up period of the study with a p-value . Since the statistical test ANCOVA was used to test the differences between treatment groups, baseline-adjusted differences were not included in the table. 95%CI were included.
